# Clinical efficacy of one-hole split endoscopy vs. unilateral biportal endoscopy for the treatment of single-segment lumbar spinal stenosis: a retrospective study with 2-year follow-up

**DOI:** 10.3389/fsurg.2025.1495741

**Published:** 2025-02-18

**Authors:** Peidong Qing, Wenlong Guo, Shiming Xie, Shengxin Zhao, Liqiang Cui, Mingfan Li, Shuangquan Gong, Youpeng Hu

**Affiliations:** ^1^ Department of Spine Surgery, Mianyang Orthopaedic Hospital, Mianyang, China; ^2^ Department of Orthopedics, Hospital of Chengdu University of Traditional Chinese Medicine, Chengdu, China

**Keywords:** one-hole split endoscope, unilateral biportal endoscope, lumbar spinal stenosis, minimally invasive surgery, spinal surgery

## Abstract

**Background:**

One-hole split endoscopy (OSE) is a novel endoscopic technique proposed by Chinese scholars in recent years. Currently, data is lacking regarding the long-term efficacy of OSE for treating lumbar spinal stenosis (LSS). This study aimed to compare the long-term efficacy of OSE and unilateral biportal endoscopy (UBE) in LSS treatment.

**Methods:**

The clinical data of 77 patients diagnosed with LSS at our hospital between January 2020 and March 2022 were retrospectively analyzed. Forty-one patients were treated with OSE, and 36 were treated with UBE. Perioperative indicators such as operation time, blood loss, fluoroscopy times, incision length, hospital stay, follow-up time, complications, and C-reactive protein level preoperatively and 3 days postoperatively were recorded. Visual analog score (VAS) and Oswestry disability index (ODI) were recorded preoperatively and at 1, 3, 6, 12, and 24 months postoperatively to evaluate pain and functional disability. The dural sac cross-sectional area (CSA), lumbar range of motion (ROM), and sagittal translation (ST) of the surgical segment were recorded preoperatively and 3 days postoperatively to evaluate lumbar stability. Clinical efficacy was assessed at the final follow-up using the modified Macnab criteria.

**Results:**

VAS and ODI scores significantly improved at each postoperative follow-up in both groups compared with preoperative values (*P* < 0.05), with no significant difference between the groups (*P* > 0.05). However, OSE had a shorter operation time, less blood loss, and shorter incision length than UBE (*P* < 0.05). Postoperative CSA was significantly increased compared to the preoperative CSA (*P* < 0.05), with no significant difference between the groups (*P* > 0.05). Postoperative ROM and ST increased; however, there was no significant difference compared to preoperative values (*P* > 0.05). The complication rates in the OSE (*n* = 2, 4.88%) and UBE (*n* = 2, 8.33%) groups were not significantly different (*x*^2^ = 0.023; *P* = 0.880). Clinical efficacy was assessed at the last follow-up using the modified MacNab criteria. Thirty-eight (92.68%) and 34 (94.44%) patients in the OSE and UBE groups, respectively, demonstrated excellent or good efficacy, with no significant difference in the efficacy rate between the groups (*x*^2^ = 0.151, *P* = 0.985).

**Conclusion:**

OSE and UBE showed satisfactory long-term efficacy and safety for LSS treatment. However, OSE has a shorter operation time, less blood loss, and shorter incision length, and can be an alternative to UBE.

## Introduction

Lumbar spinal stenosis (LSS) is a syndrome of pain in the buttocks or lower extremities with intermittent claudication, possibly with or without low back pain (1). Most LSS cases present as acquired degenerative stenosis caused by spinal degeneration or after surgery or infection (2), which can lead to persistent chronic pain and disability, severely affecting the quality of life, activities of daily living, and function (3). In patients with LSS in whom conservative treatment fails, surgery remains an inevitable intervention strategy (4). Traditional open surgery, including unilateral laminotomy, bilateral laminotomy, and spinous osteotomy, are all classic and effective surgical procedures for the treatment of LSS (5). Notably, injuries associated with open surgery cannot be ignored. Intraoperative stripping of large areas of paravertebral muscle and removal of the facet joints and vertebral plates will lead to various postoperative complications (6, 7), such as persistent low back pain, lumbar instability, and muscle denervation (8). The development of spinal endoscopy seems to compensate for the limitations of open surgery. The unilateral biportal endoscopy (UBE) has been widely used in the treatment of LSS and has the advantages of flexible operation, wide surgical vision, less tissue damage, and superior clinical efficacy (9, 10). The development of UBE technology effectively overcomes the problems of limited access and limited surgical vision in single-portal endoscopic techniques and traditional microscopic surgery (11).

One-hole split endoscopy (OSE) was first proposed and applied in the clinic by Chinese scholars in 2019 (12). OSE is similar to UBE in that it has an operating channel and an observation channel. The difference is that the two channels of the OSE technique are located in the same surgical incision, and this configuration allows the surgical instruments and the endoscopic system to rotate and oscillate independently and flexibly without being constrained by a fixed conduit (13). This provides the OSE technique with greater operational flexibility and a wider surgical view. OSE, a new method that combines the convenience of single-portal endoscopy with the advantages of a wide surgical view of biportal endoscopy, is currently used by spine surgeons to treat LSS in China. However, studies on the long-term efficacy of OSE for the treatment of LSS are lacking. Therefore, this study retrospectively analyzed the long-term efficacy of the OSE technique in the treatment of LSS and compared it with the UBE technique undertaken at the same time, aiming to provide reference and guidance for the application of OSE in clinical practice.

## Methods

### Participant data

The clinical data of 77 patients admitted to our hospital between January 2020 and March 2022 were retrospectively analyzed. These patients were diagnosed with single-segment LSS by a spine surgeon with extensive experience (>5000 spine surgeries) and treated with OSE or UBE. The selection of the surgical procedure was based on the surgeon's discretion and patient choice. Both surgical procedures were fully explained to the patient before surgery, and informed consent was obtained from the patient.

The inclusion criteria for this study were as follows: (1) low back pain or radicular leg pain with or without intermittent neurological claudication; (2) computed tomography (CT) or magnetic resonance imaging (MRI) showing LSS (central, lateral, or mixed stenosis); (3) no improvement after ≥3 months of formal conservative treatment; and (4) follow-up time ≥2 years with complete data.

The exclusion criteria were as follows: (1) lumbar tuberculosis, tumor, trauma, or infection; (2) surgery exceeding one surgical level; and (3) previous lumbar surgery.

## Surgical procedure

### OSE group

**Figure 1:**
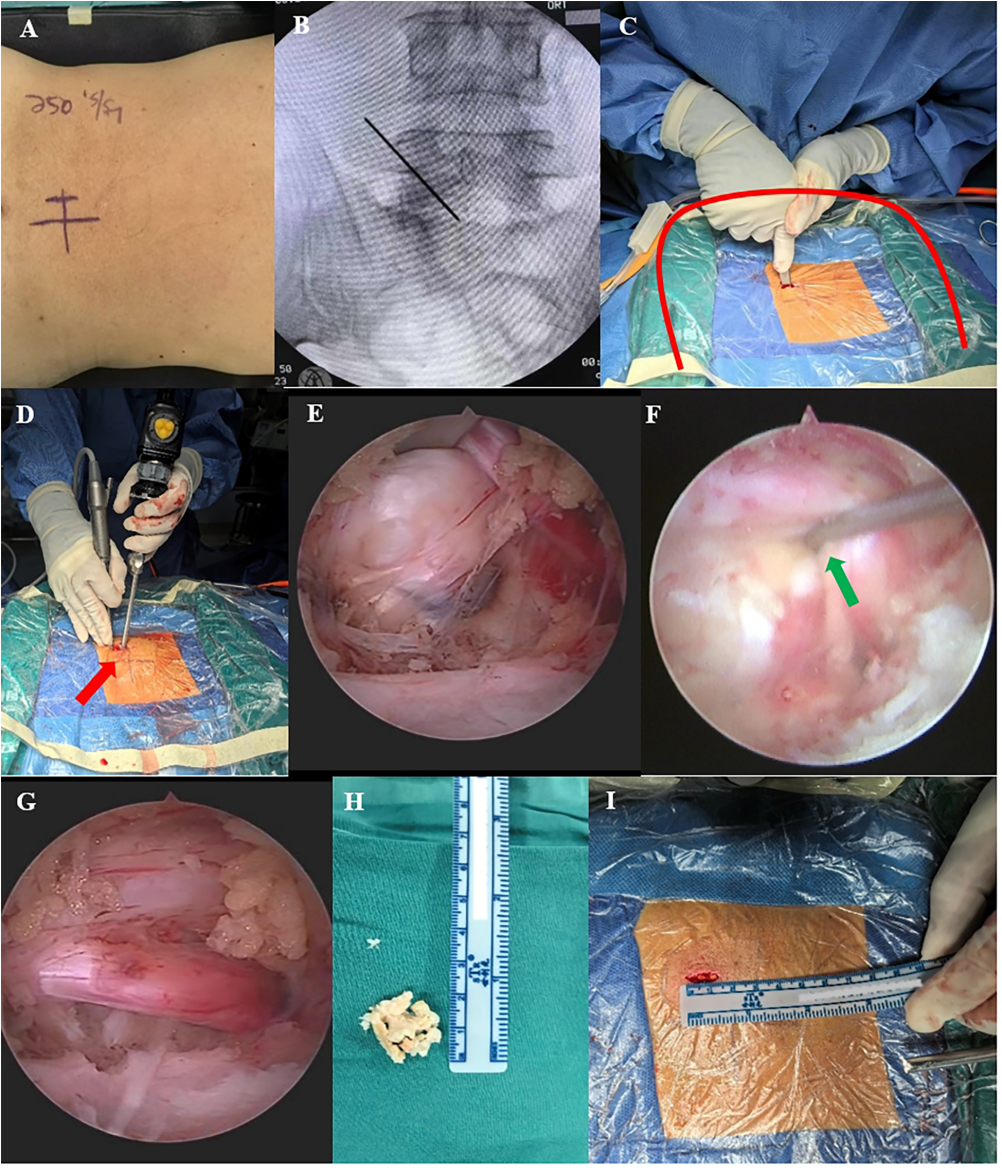
Intraoperative OSE images. **(A,B)** Preoperative C-arm fluoroscopy was performed to localize the target segment. **(C)** Use of the dilator to establish surgical access (the red curve shows the U-shaped channel created by spreading the towel, which facilitates the outflow of the irrigation solution). **(D)** Manipulation and observation in one hole (red arrow). **(E,F)** Endoscopy showing a massive lumbar disc herniation, with gentle retraction using a nerve retractor to remove the herniation (green arrow shows nerve retractor). **(G)** Completely decompressed nerve root with reduced nerve root tension and restoration of spontaneous pulsation. **(H)** Removed discs and soft tissue. **(I)** Length of OSE surgical incision.

The patient was placed in the prone position on a radiolucent surgical table under general anesthesia, with the abdomen in suspension and the lumbar bridge slightly flexed to open the surgical segment slightly. Consider the left surgical approach as an example. After routinely sterilization and towel application, a sheet was used to create a U-shaped drainage channel in the surgical area to facilitate irrigation fluid outflow. The endoscope, radiofrequency electrode, grinding drill, and perfusion system were then connected. The surgical segment was localized under C-arm fluoroscopy, and a longitudinal incision approximately 1.5 cm long was made 2 cm to the left of the intersection of the surgical segment and the spinous process, followed by sequential cutting of the skin, subcutaneous tissue, and deep fascia. A dilator was used to gradually expand the soft tissue to the surface of the vertebral plate, and the OSE endoscopic system and surgical instruments were then placed. The soft tissues of the vertebral plate surfaces were cleared using radiofrequency electrodes to expose the lower edge of the plate in the upper vertebrae, upper edge of the plate in the lower vertebrae, medial edge of the facet joint closure, and lateral edge of the base of the spinous process. The inferior edge of the superior vertebral plate and the superior edge of the inferior vertebral plate were partially removed using a dynamic drill to expose the attachment points of the ligamentum flavum, which was gently stripped away from the dural sac, exposing the dural sac, intervertebral discs, and nerve roots. The dural sac was gently retracted using a nerve retractor to completely remove herniated disc tissue and hypertrophied posterior longitudinal ligament tissue, and adequate decompression was performed around the nerve root canal. Complete decompression was confirmed when decreased nerve root tension and spontaneous pulsations were observed. Finally, meticulous and sufficient hemostasis was performed using a radiofrequency electrode, the endoscopic system was removed, and the wound was sutured and covered with sterile dressing (Figure 1).

### UBE group

**Figure 2:**
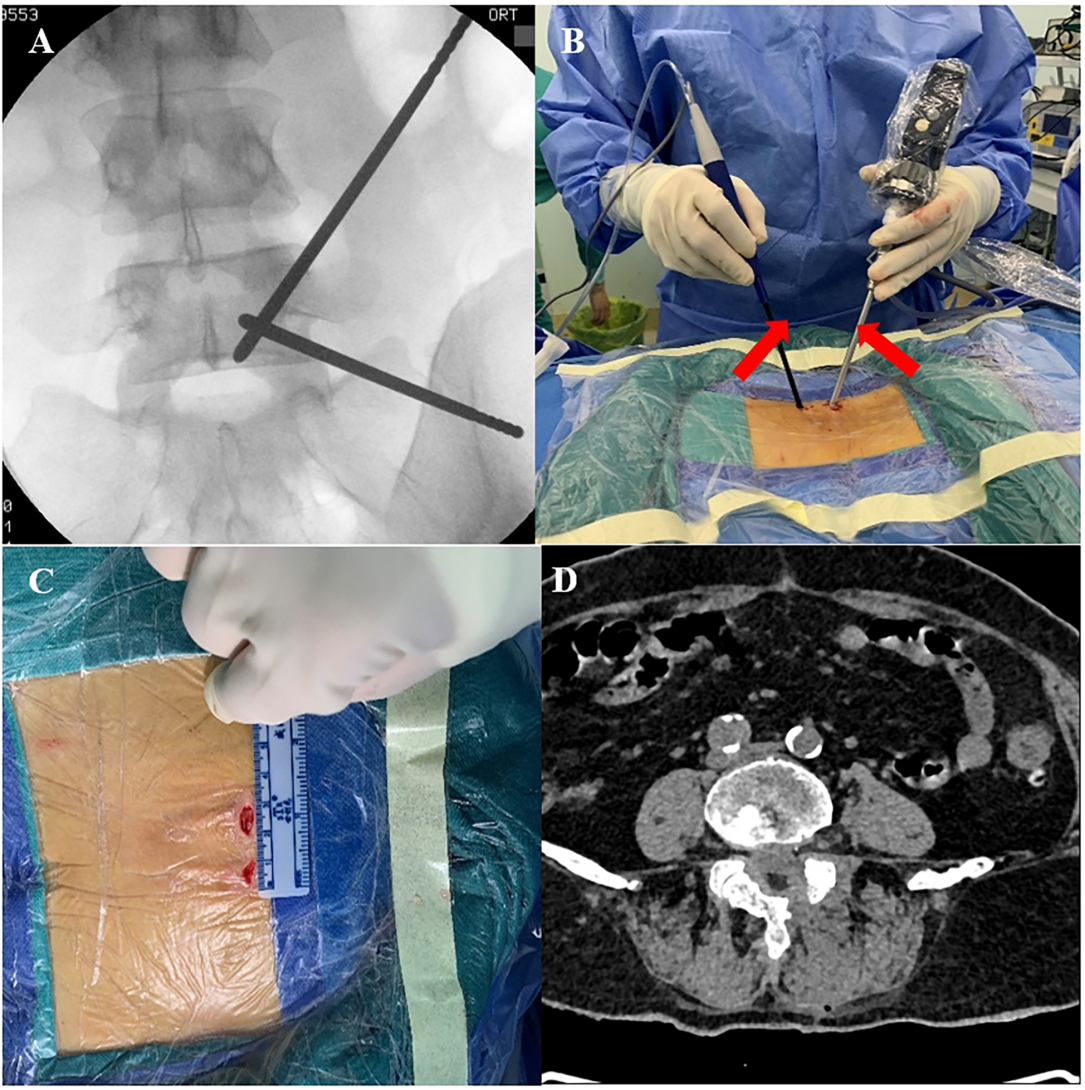
Intraoperative UBE. **(A)** Preoperative C-arm fluoroscopy to localize the target segment. **(B)** Manipulation and observation in two portals (red arrows). **(C)** Length of UBE surgical incision. **(D)** Postoperative CT showing complete decompression of the spinal canal.

The surgical segment was localized under C-arm fluoroscopy, and the intersection point was made with the line connecting the surgical segment and medial aspect of the left upper and lower pedicles. Two transverse surgical incisions of approximately 1.5 cm in length were made 1.5 cm above and below the intersection point, with one near the cephalad side as the observation portal and the other near the caudal side as the operation portal. The soft tissues were gradually expanded to the surface of the vertebral plate using a dilator, and the endoscopic system and surgical instruments of the UBE were placed. The remaining steps were identical to those in the OSE group (Figure 2).

### Postoperative treatment

All patients were administered postoperative medication such as mecobalamine to nourish their nerves and mannitol to reduce nerve root edema. On the second postoperative day, they were encouraged to stand out of bed with the help of waist support. Patients were instructed to wear waist support for 4 weeks, avoid strenuous exercise during this period, and undergo regular review with radiography, CT, and MRI.

### Clinical data collection

Perioperative-related indicators were recorded, such as operation time, blood loss, wound length, fluoroscopy time, hospital stay, follow-up time, complications, and preoperative and 3-day postoperative CRP levels.

The VAS and ODI scores were recorded preoperatively and at 1, 3, 6, 12, and 24 months postoperatively to assess pain and functional improvement.

**Figure 3:**
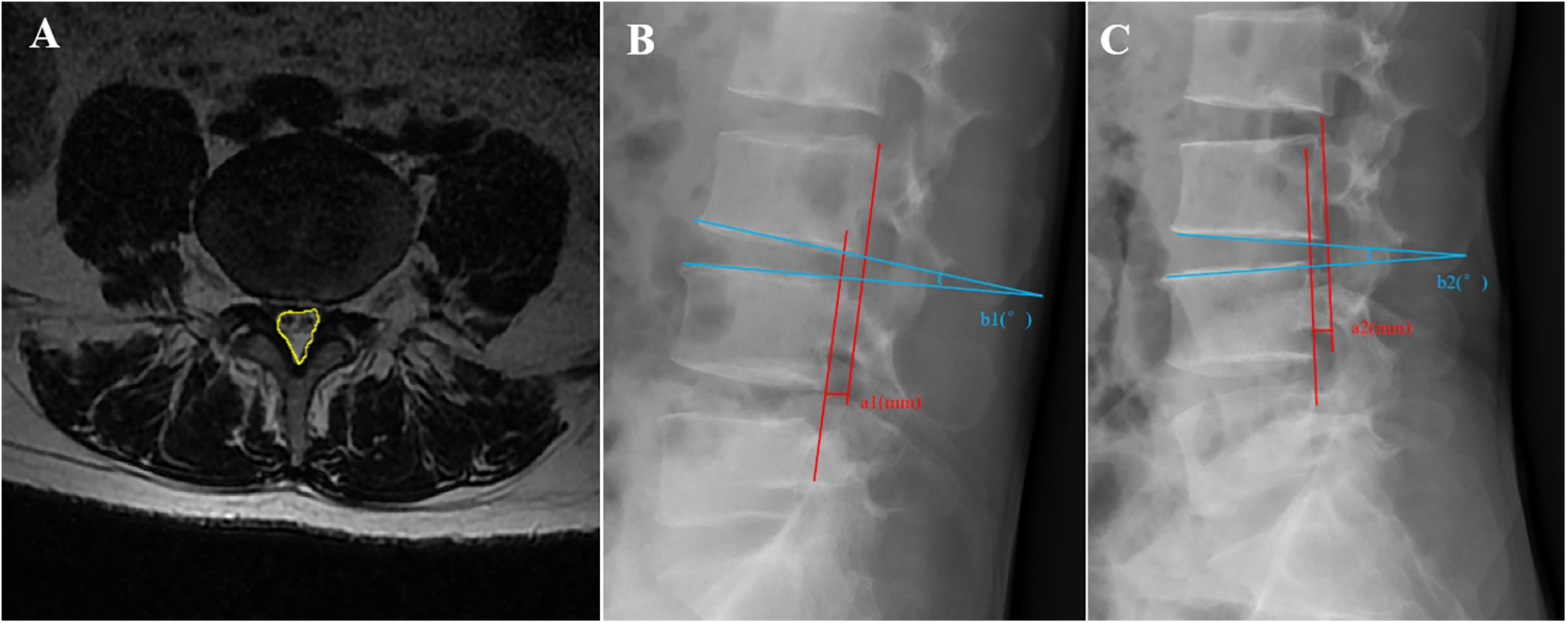
Schematic of the imaging measurements. **(A)** Measurement of the dural sac cross-sectional area (CSA) (yellow area). **(B,C)** Measurement of the lumbar range of motion (ROM) and sagittal translation (ST). ROM = b2 − b1, ST = |a2 − a1|.

The dural sac cross-sectional area (CSA), range of motion (ROM) of the surgical segment, and lumbar sagittal translation (ST) of the responsible segment were recorded preoperatively and 3 days postoperatively. Specifically, the dural sac CSA (Figure 3A) was measured on axial films of T2-weighted MRIs of the surgical segments of the patients using ImageJ 1.54 software (National Institutes of Health, USA) (14), with a total of three measurements for each responsible interval, which were averaged to assess the degree of spinal canal decompression for the two groups**.** The ROM and ST of the surgical segment were measured using lumbar dynamic-position radiography (Figures 3B,C) to assess the stability of the surgical segment. The lumbar spine was defined as unstable when the ROM was ≥15° or ST was ≥3 mm (15). Clinical outcomes of patients were assessed at the last follow-up using the modified Macnab criteria.

### Statistical analysis

SPSS 26.0 (IBM SPSS Statistics for Windows, Armonk, New York, USA) was used to perform the statistical analyses. Continuous variables that conformed to a normal distribution are expressed as mean ± standard deviation and evaluated using Student's *t*-test. Non-normally distributed continuous data were assessed using the Mann–Whitney *U* test. Categorical variables are expressed as frequency or percentage and were evaluated using the chi-square test or Fisher exact test. Statistical significance was set at *P* < 0.05.

## Results

### Baseline characteristics

Seventy-seven patients (36 men and 41 women) were included in this study. Forty-one patients (21 men and 20 women) were included in the OSE group, with a mean age of 66.51 ± 5.62 years. Thirty-six patients (15 men and 21 women) were included in the UBE group, with a mean age of 65.44 ± 6.51 years. Detailed patient characteristics are shown in Table 1. There was no statistically significant difference between the two groups in age, sex, BMI, disease course, hypertension, diabetes, stenotic type, and target level (*P* > 0.05).

**Table 1:** Demographic data for the two groups.

	OSE (*n* = 41)	UBE (*n* = 36)	*P* value
Age (years)	66.51 ± 5.62	65.44 ± 6.51	0.343
Gender (*n*)			0.402
Male	21	15	
Female	20	21	
BMI (kg/m^2^)	23.46 ± 2.65	23.16 ± 2.33	0.502
Disease course (months)	24.54 ± 25.73	21.44 ± 23.39	0.429
Stenotic type (*n*)			0.171
Central canal stenosis	14	6	
Lateral recess stenosis	16	15	
Mixed stenosis	11	15	
Target level (*n*)			0.286
L3/4	7	4	
L4/5	20	24	
L5/S1	14	8	
Hypertension (*n*)	18	14	0.656
Diabetes (*n*)	17	12	0.463

OSE, one-hole split endoscope; UBE, unilateral biportal endoscope; BMI, body mass index.

### Perioperative outcomes

Operation time, blood loss, and incision length were significantly superior in the OSE group than in the UBE group (*P* < 0.05), whereas there were no significant differences in hospital stay, fluoroscopy time, and follow-up duration between the two groups (*P* > 0.05). The CRP level was significantly increased in both groups postoperatively (*P* < 0.05), whereas there was no significant difference in the comparison between the two groups both preoperatively and postoperatively (*P* > 0.05). There were two (4.88%) complications in the OSE group and three (8.33%) complications in the UBE group. However, there was no significant difference in the complication rate between the two groups (*P* > 0.05) (Table 2).

**Table 2:** Comparison of operative outcomes between the two groups.

	OSE (*n* = 41)	UBE (*n* = 36)	*P* value
Operation time (mins)	71.95 ± 15.23	76.94 ± 10.15	0.002*
Blood loss (ml)	63.27 ± 17.28	68.14 ± 24.77	0.024*
Incision length (cm)	1.19 ± 0.33	2.81 ± 0.51	0.005*
Hospital stay (days)	6.32 ± 1.69	6.47 ± 1.40	0.143
Fluoroscopy times	1.88 ± 0.64	2.08 ± 0.84	0.189
Follow-up time (months)	27.88 ± 3.87	28.22 ± 4.39	0.214
CRP (mg/L)			
Pre-op	5.16 ± 2.33	5.03 ± 2.79	0.998
Post 3d	24.51 ± 8.02	23.77 ± 13.64	0.175
*P*1 value	<0.001*	<0.001*	

*P* value indicates a comparison between the two groups.

*P*1 value indicates a comparison with the preoperative value.

OSE, one-hole split endoscope; UBE, unilateral biportal endoscope; CRP, C-reactive protein.

* Indicates a significant difference.

### Clinical outcomes

**Figure 4:**
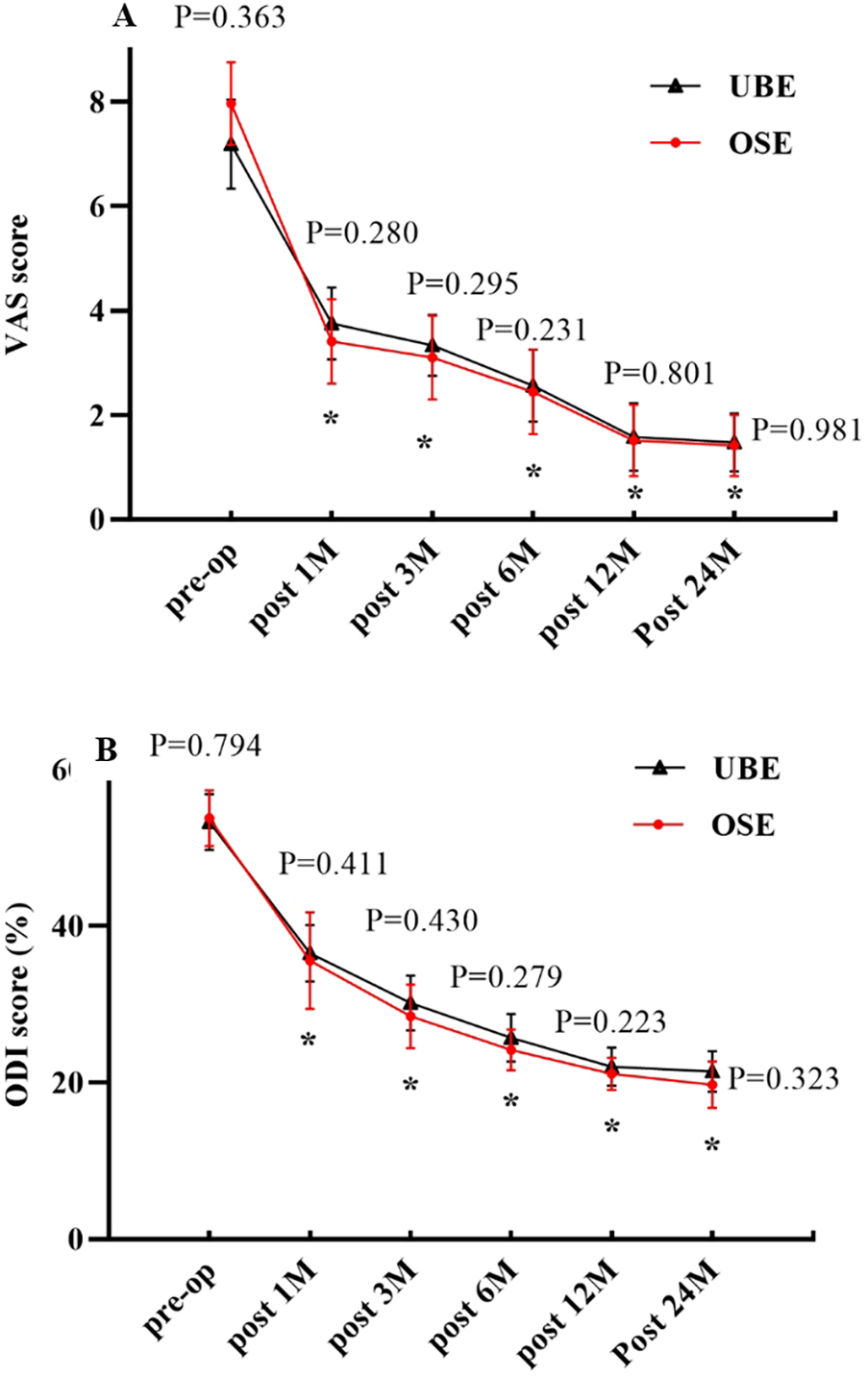
**(A)** Changes in VAS scores over time. **(B)** Changes in ODI scores over time. *P* indicates a comparison between the two groups. *indicates comparison with the preoperative value.

The preoperative VAS and ODI scores were comparable between the two groups (*P* > 0.05). VAS scores in the OSE and UBE groups significantly decreased from 7.97 ± 0.79 and 7.19 ± 0.86 preoperatively to 3.41 ± 0.81 and 3.75 ± 0.69 at 1 month postoperatively (*P* < 0.05). There was a significant decrease in VAS scores at 3, 6, 12, and 24 months postoperatively compared to preoperatively (*P* < 0.05). The ODI scores in the OSE and UBE groups decreased from 53.76 ± 3.58 and 53.25 ± 3.56 preoperatively to 35.56 ± 6.16 and 36.53 ± 3.6 at 1 month postoperatively, and the difference was statistically significant (*P* < 0.05). There was a significant decrease in ODI scores at 3, 6, 12, and 24 months postoperatively compared to preoperatively (*P* < 0.05). There were no significant differences in the VAS and ODI scores between the two groups at each postoperative follow-up time point (*P* > 0.05) (Figures 4A,B). At the last follow-up, 38 patients in the OSE group had excellent or good outcomes, with an overall efficacy rate of 92.68%. Thirty-four patients in the UBE group showed excellent or good outcomes, with an overall efficacy rate of 94.44%. There was no significant difference in the efficacy rates between the two groups (*x*^2^ = 0.151, *P* = 0.985; Table 3).

**Table 3:** Modified macnab criteria between the two groups.

	Cases	Excellent	Good	Fair	Poor	Rate (Excellent and Good)
OSE	41	25	13	2	1	38 (92.68%)
UBE	36	20	14	1	1	34 (94.44%)
*x* ^2^	0.151					
*P* value	0.985					

OSE, one-hole split endoscope; UBE, unilateral biportal endoscope.

### Radiographic outcomes

**Figure 5:**
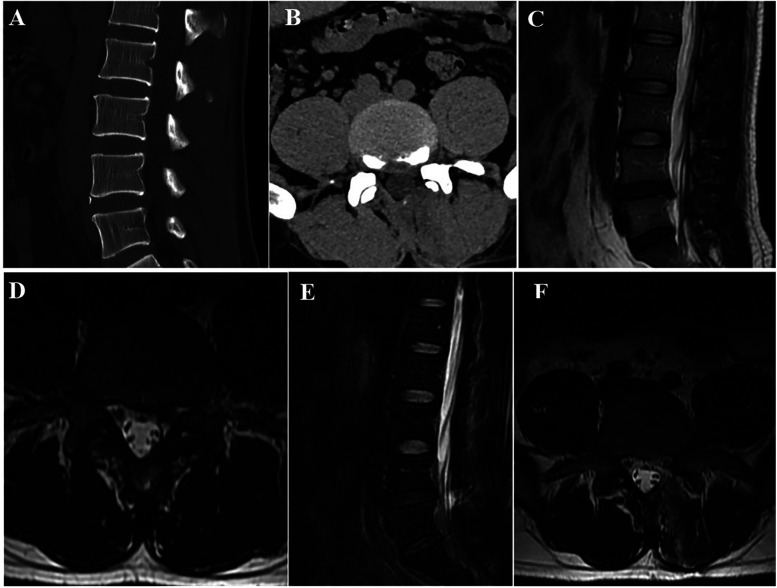
Radiographic data of a patient treated with OSE. **(A,B)** Transverse and sagittal lumbar CT revealed LDH with severe lumbar lateral recess stenosis at L4/5. **(C,D)**. Lumbar MRI showed the same results as the CT. **(E,F)** Lumbar MRI after OSE showed that the lumbar lateral recess stenosis was completely opened.

The radiographic outcomes of the OSE and UBE groups are shown in Table 4. There was no significant difference between CSA, ROM, and ST in both groups preoperatively (*P* > 0.05). Compared with the preoperative values, CSA significantly improved postoperatively in both groups (*P* < 0.05); however, there was no significant difference in the changes between the two groups (*P* > 0.05). The postoperative ROM was 9.70 ± 1.67° and 10.63 ± 1.84°, respectively. There was no significant difference between the two groups (*P* > 0.05), and both were less than 15°. Postoperative ST was 1.51 ± 0.68 mm and 1.58 ± 0.65 mm, respectively, and there was no significant difference between the two groups (*P* > 0.05), and both were less than 3 mm. Postoperative ROM and ST were within normal limits (ROM ≤ 15° and ST ≤ 3 mm), and no lumbar instability was observed (a typical case of OSE is shown in Figure 5).

**Table 4:** Comparison of radiographic data between the two groups.

	OSE (*n* = 41)	UBE (*n* = 36)	*P* value
CSA (mm^2^)			
Pre-op	81.05 ± 8.38	81.27 ± 9.77	0.844
Post 3d	157.24 ± 18.83	162.11 ± 17.63	0.859
*P*1 value	<0.001*	<0.001*	
ROM (°)			
Pre-op	8.48 ± 1.05	8.54 ± 1.03	0.844
Post 3d	9.70 ± 1.67	10.63 ± 1.84	0.928
*P*1 value	0.084	0.128	
ST (mm)			
Pre-op	1.37 ± 0.49	1.53 ± 0.51	0.126
Post 3d	1.51 ± 0.68	1.58 ± 0.65	0.801
*P*1 value	0.262	0.701	

*P* value indicates a comparison between the two groups.

*P*1 value indicates a comparison with the preoperative value.

OSE, one-hole split endoscope; UBE, unilateral biportal endoscope.

* Indicates a significant difference.

## Complications

All patients completed the surgery, with two complications in the OSE group and three in the UBE group. There was no significant difference in the complication rate between the two groups (4.88% vs. 8.33%, *P* = 0.880; Table 5). Two patients demonstrated temporary postoperative hyperalgesia, which improved after treatment with nutritive nerve medication. Two other patients presented with intraoperative dural tears, which were compressed intraoperatively with gelatin sponges since the tears were small, and there was no postoperative cerebrospinal fluid leakage. In addition, one patient demonstrated residual symptoms that improved with conservative treatment.

**Table 5:** Postoperative complications between the two groups.

	OSE (*n* = 41)	UBE (*n* = 36)	*P* value
Dural tear	1 (2.44%)	1 (2.78%)	1.000
Residual symptoms	0	1 (2.78%)	0.468
Temporary hyperalgesia	1 (2.44%)	1 (2.78%)	1.000
Wound infection	0	0	NA
Total	2 (4.88%)	3 (8.33%)	0.880

NA, not applicable; OSE, one-hole split endoscope; UBE, unilateral biportal endoscope.

## Discussion

LSS is a prevalent cause of chronic, insidious low back pain, especially in elderly patients (16). It is estimated that approximately 103 million people worldwide are affected by LSS (17). With disease progression, LSS has a serious impact on the quality of life of patients (18), which undoubtedly imposes a huge burden on the world's public health system. A 10-year prospective study suggested that surgical treatment was aggressive and meaningful for patients with LSS (19). Over the past few decades, conventional open laminectomy has become a standard and effective treatment approach for LSS (5); however, the complications associated with this technique should not be overlooked (20). In particular, stripping of muscles and ligaments at the back of the spine may result in persistent postoperative low back pain and muscle atrophy (21), and the loss of bony structures at the back of the spine may lead to postoperative lumbar instability; in severe cases, additional fusion surgery may be required (22). Minimally invasive spinal endoscopy has been developed to minimize injuries associated with surgery. UBE, introduced by Kambin and Sampson in 1986, has re-entered the limelight in recent years with the impetus and refinement of Korean physicians (23, 24). UBE is now widely used in the treatment of LSS (25, 26) and provides the following advantages. First, UBE offers both the flexibility of open surgery and the enhanced clarity of vision of minimally invasive surgery. Moreover, the surgical approach and view are similar to those of open surgery and use the principles of arthroscopic triangulation, which is easier for beginners to study. Third, the contralateral sublaminar space and intervertebral foraminal area can be easily observed by moving the endoscope without additional incisions or position adjustments, making it easier to perform contralateral decompression. Fourth, UBE can be performed using open surgical instruments, which could reduce the economic burden of performing this technique in primary hospitals. Previous studies have demonstrated the satisfactory surgical efficacy of UBE in the treatment of LSS and its ability to achieve a positive clinical outcome (27, 28).

OSE is an emerging spinal endoscopic technique based on innovative advances in UBE technology (12). Currently, it has been applied to the treatment of various degenerative spinal diseases such as LDH (29), LSS (30), thoracic ligamentum flavum ossification (31), and lumbar spondylolisthesis (32).

Similar to UBE, OSE provides two channels, a viewing channel and an operating channel, with the difference that the two channels of OSE are in the same soft incision, allowing unrestricted movement of the endoscope and surgical instruments through a single port (29). This configuration gives OSE unique advantages over UBE. Furthermore, UBE is performed through two portals; therefore, surgical instruments and endoscopes need to be placed at a “V” angle, whereas OSE is free of similar limitations, effectively avoiding blind spots caused by the UBE technique. Therefore, OSE is effective in minimizing nerve root and dural sac damage in patients with severe spinal stenosis. Additionally, OSE is performed within a portal, avoiding the possibility of misplacement of surgical instruments due to misalignment, making it more efficient when performing decompression and hemostasis. Spine surgeons with UBE experience may have a shorter learning curve when performing OSE and may be able to treat LSS more proficiently. Adequate practical experience will help beginners overcome their learning curve more quickly. Nonetheless, when dealing with patients with severe spinal stenosis, decompression maneuvers with OSE should be performed more cautiously to avoid damage to the nerve roots and dural sac caused by retraction of the conventional open instrumentation (12).

In this study, both the OSE and UBE groups showed positive clinical outcomes in the treatment of LSS, with both groups showing significant improvements in VAS and ODI scores postoperatively and maintaining favorable outcomes until 2 years postoperatively, suggesting that both techniques are effective in alleviating pain and dysfunction associated with LSS. However, there were no significant differences between the two groups at different postoperative follow-up times. Although the two techniques have similar clinical efficacy in the treatment of LSS, OSE still presents unique characteristics. Compared with UBE, OSE has a shorter operation time, less blood loss, and shorter incision length. Operation time is positively associated with postoperative complications such as infection, blood transfusion, and prolonged hospital stay (33), and a reasonable reduction in operation time will help reduce the occurrence of postoperative complications. The positioning of the vertebral plate during OSE is easier and quicker, and there is no restriction of the “V” angle, which makes the operation more comfortable and may reduce the operation time to some extent.

CRP is an acute inflammatory serum marker; however, it is not a specific marker of tissue damage caused by surgery (34). Nevertheless, there was no significant difference in CRP levels between the two groups postoperatively, suggesting that tissue destruction was similar in both groups. Choi (35) indicated that continuous irrigation during endoscopic procedures would help remove inflammatory debris during the procedure, thus helping to reduce the CRP level.

Previous studies have shown that patients with LSS undergoing surgical treatment could expect an increase in CSA if decompression was sufficient to relieve early postoperative symptoms (36). Sufficient spinal canal decompression and lumbar stability are critical factors for the prognosis of patients with LSS. In this study, both groups demonstrated a significant increase in postoperative CSA without compression of the nerve roots or dural sac, indicating that both surgical approaches had adequate decompression benefits.

Partial resection of the facet joints is inevitable for both OSE and UBE to create surgical access and achieve adequate decompression; however, resection of the small joints will potentially affect lumbar segmental stability (37). A finite element analysis showed that partial resection of the small joints increased the ROM of the lumbar spine (38). In this study, there was an increase in postoperative ROM compared with preoperative ROM in both groups; however, with no significant difference, there was also no significant difference in the postoperative comparison between the two groups. In addition, no patients were observed with lumbar instability postoperatively in this study, suggesting that OSE and UBE had no significant effect on postoperative lumbar stability in patients with LSS, which was similar to a previous study (7).

Medical dural tear is the most common complication of endoscopic surgery (39), with an overall rate of approximately 2.7% (range, 0%–8.6%). In contrast, the incidence of dural tears in patients with LSS is even higher, at approximately 3.7% (40). The incidence of dural tears in the OSE and UBE groups in this study was 2.44% and 2.78%, respectively, which was similar to the results of previous studies (18). Although OSE provides a clearer surgical view than open surgery, saline irrigation during the procedure can enlarge the gap between the ligamentum flavum and dural sac to make its separation safer. However, there are several possible reasons for dural tears. First, in the OSE procedure, the injected saline squeezed both sides of the dura mater, causing the area to fold. The central region may be damaged during ligamentum flavum resection. Second, when using high-speed drills, fibrous bands and vascular bundles around the periphery of the dura mater may stretch around the drill neck, causing greater tears (40). Third, OSE does not require retraction of anatomical structures to expose the dura mater, which is a major difference from other surgical techniques. For beginners, gentle operation and careful hemostasis can help minimize the possibility of postoperative complications when faced with severe adhesions between the dural sac and nerve roots. In addition, preoperative preparation of an individualized decompression plan will help achieve a satisfactory clinical outcome.

The current study had some limitations. First, the same spine surgeon performed all procedures, and there may be some bias. Second, the study was retrospective and had a small sample size with unavoidable confounding factors between the included groups. Further validation of these findings is expected from future large-scale randomized controlled trials.

## Conclusion

Both OSE and UBE have satisfactory long-term outcomes in the treatment of LSS. However, OSE provides shorter operation time, less blood loss, and shorter surgical incision, which can serve as an alternative surgical procedure to UBE.

## Data Availability

The original contributions presented in the study are included in the article/Supplementary Material, further inquiries can be directed to the corresponding author.
